# Has a national programme to expand the nursing workforce in England been a success? A mixed methods study

**DOI:** 10.1016/j.ijnsa.2026.100735

**Published:** 2026-11-05

**Authors:** Joanne Turnbull, Lucina Rolewicz, William Palmer, Julia Philippou, Anne Marie Rafferty, Matthew Williams, Jane Prichard, Ourega-Zoé Ejebu, Jane Frankland, Jaimie Ellis, Iain Atherton, Michelle Jamieson, Jane Ball

**Affiliations:** a School of Health Sciences, University of Southampton, UK; b The Nuffield Trust, London, UK; c Florence Nightingale School of Nursing and Midwifery, King's College, London, UK; d Institute for Employment Studies, Brighton, UK; e School of Health and Social Care, Edinburgh Napier University, Edinburgh, UK

**Keywords:** Nursing staff, Health workforce, Healthcare policy, Health care systems, National health service

## Abstract

**Background:**

Many countries face long-term nursing workforce challenges, with registered nurse shortages placing pressure on care delivery and compromising patient safety and outcomes. In England, a substantial shortfall prompted the government’s ‘N50k’ programme, which aimed to increase National Health Service nurse numbers by 50,000 between 2019 and 2024 by expanding domestic supply and international recruitment and improving retention.

**Objective:**

To describe and evaluate the efficacy of a national registered nurse workforce expansion policy and its component strategies.

**Design:**

Mixed methods evaluation.

**Setting(s):**

England, UK.

**Methods:**

Mapping N50k programme objectives, proposed interventions and activities from internal programme documents; analysis of data from before and during N50k period (2019–2024) on: NHS joiners and leavers (31 m NHS staff records), and student enrolments and qualifiers between 2014/15 to 2023/24; a survey of all 210 NHS trusts in 2023 (55 % trusts participated) and two qualitative case studies (31 interviews in total).

**Results:**

The 50,000 target was met early. The N50k programme, and associated investment, accelerated substantial structural and operational changes, notably rapid expansion of international recruitment, and financial support for employers offering nursing apprenticeships. 40,531 registered nurses joined NHS from non-EU/non-UK countries (January 2020 to June 2023) accounting for 43.7 % of all new joiners, over double the initial N50k target of 18,400. Investment in pre-arrival, onboarding and training support enabled smoother transition into roles. Nurse retention improved but it was unclear whether this was a result of programme impact, wider context or initiatives already in place. 83 % of respondents from NHS trusts agreed that retention rates had improved at their trust since 2019, but only 30 % agreed N50k had a significant effect on the trust’s ability to retain staff. Retention interventions focused on flexible working, professional development and early-career support. Organisational culture and supportive teamwork were seen as key retention factors.

**Conclusions:**

This programme - driven by a political manifesto commitment - succeeded in creating targeted investment and focussed effort into growing the nursing workforce. However, once the target had been achieved, investment has largely been discontinued. Improvements designed to increase domestic supply and increase retention were at risk of being discontinued. Longer-term commitment to nursing workforce development and growth is needed if domestic supply is to be increased so the NHS can be less reliant on international recruitment in the future. A sustained focus on retaining nurses is critical to maximising the recruitment success of the N50k programme.

**Registration:**

Not registered.

**What is already known**

• Shortages of registered nurses is associated with poorer patient safety and outcomes.
• International recruitment has long been essential for workforce growth.
• Retention challenges are well documented across the international literature.

**What this paper adds**

• The first comprehensive mixed-methods evaluation of a national policy combining nurse recruitment, and retention.
• The N50k programme achieved rapid workforce growth primarily through international recruitment, while producing limited sustained expansion in domestic supply.
• The findings show that target-led workforce growth is unlikely to be sustained without long-term investment in domestic education, retention and support for internationally recruited nurses.

## 1 Background

The global nursing workforce grew from 27.9 million in 2018 to 29.8 million in 2023 but there is wide variation in the availability of registered nurses between countries (World Health Organization, 2025) and ahead, long-term workforce challenges are predicted. Ageing populations and increasing healthcare need are intensifying health service demand. Additionally, the registered nursing workforce is ageing, particularly in high-income countries (Tamata and Mohammadnezhad, 2023). Nursing shortages are associated with poorer patient safety and outcomes and increased pressure on staff, making jobs less attractive to new or existing staff (Nursing and Midwifery Council, 2021; Dall'Ora et al., 2022; House of Commons Health and Social Care Committee, 2021). In England, persistent undersupply of nurses relative to demand led to a significant shortfall in the National Health Service (NHS) (Buchan et al., 2019; House of Commons Library, 2020). In 2019, estimates indicated a potential shortfall of 108,000 by 2029 without intervention (Beech et al., 2019), with particularly notable vacancies in community, learning disability and mental health nursing (Buchan et al., 2020).

In December 2019, the Conservative government committed to increasing the number of registered nurses working in the NHS in England by 50,000 during the 2019–2024 parliamentary period (Conservative and Unionist Party, 2019). The programme was implemented amid policy and system pressures that further weakened domestic nursing supply and increased reliance on international recruitment. Following the 2016 European Union referendum (‘Brexit’), uncertainty contributed to declining recruitment from the European Union/European Economic Area (EU/EEA). These effects intensified after free movement between the UK and the EU ended in 2021, shifting NHS recruitment in England towards wider global labour markets (Dayan et al., 2025). The removal of the NHS bursary for new nursing students in England in 2017 transferred tuition and living costs onto students through repayable loans (Department of Health and Social Care, 2017). Undergraduate nursing applications subsequently declined, despite a temporary pandemic-related rise (University College and Admissions Service, 2022). COVID-19 compounded workforce pressures through staff sickness and redeployment (NHS England and NHS Improvement, 2020) as well as disrupting student education and clinical placements (Brett et al., 2024). Exposure to high workload, infection risk and patient deaths increased stress and burnout, strengthening the case for workforce expansion alongside retention interventions (Couper et al., 2022; Royal College of Nursing, 2022).

Referred to in this paper as the ‘N50k programme’ (Department of Health and Social Care, 2022), the programme comprised bundles of policies organised across three workstreams: increasing the domestic supply of new nurses, expanding international recruitment and reducing the outflow of existing nurses through improved retention.

*Domestic supply.* A sustainable workforce pipeline relies on attracting people into the profession, entry routes, training and clinical placement capacity, and course completion rates (Palmer et al., 2023). In England, the main pathways to becoming a registered nurse include undergraduate nursing degrees, graduate entry programmes, and registered nurse degree apprenticeships (Department for Education, 2024). Nursing associates and associate practitioners (roles that bridge the gap between healthcare assistants and registered nurses) can become registered nurses through "top-up" routes, usually a shortened nursing degree or degree-level apprenticeship. The N50k programme aimed to expand numbers across all routes. Supply is also constrained by the impact of course attrition (Brook et al., 2019). An earlier large-scale project identified financial pressures, student confidence, academic attainment and clinical placement challenges as major causes of attrition (Health Education England, 2018).

*International recruitment.* The NHS has long recruited internationally to address shortages, particularly from India, the Philippines and Nigeria (NHS England, 2023; Nursing and Midwifery Council, 2025). Since domestic expansion requires several years, it offers rapid workforce growth but increases reliance on lower-resource countries. In 2022/23, over 40 % of new registrants were educated in low- or lower-middle-income countries, including over 6000 from countries with critical health worker shortages (Buchan et al., 2023). The UK Code of Practice and WHO Global Codes restricts active recruitment from countries on its red list (countries identified as having significant health workforce shortages) unless recruitment occurs through government-to-government agreement; individuals from these countries may apply independently. Ethical recruitment also requires fair employment, protection from recruitment fees, effective induction, and avoiding harm to source-country health systems (Department of Health and Social Care, 2025a; World Health Organization, 2010).

*Retention.* The reasons for nurse turnover are well researched and new entrants and older nurses are at greatest risk of leaving (Ejebu et al., 2024). N50k intervnetions and activities built upon previous initiatives and policy, for example the NHS Nursing Retention Programme (NHS England, 2021), rather than operating as a standalone intervention. The N50k programme aimed to improve the experience and retention of nurses in the NHS through ‘policy bundles’ rather than single interventions, which typically have the greatest impact (NHS England, 2021).

The aim of our research was not to evaluate if the N50k programme met the target of expanding the workforce by 50,000 nurses by March 2024. Indeed, in November 2023, the previous government announced the programme reached the target early (Department of Health and Social Care, 2023). Instead, we evaluated how well the multi-strand initiatives in the three N50k workstreams worked, and what can be learned to inform future strategies for the recruitment and retention of nurses. This paper draws on mixed-methods data to examine ‘what worked’ against the key criteria of increasing domestic supply, international recruitment, retention rates, and we examine how the programme could be improved.

## 2 Methods

### 2.1 Study design

The study objectives and methods included in this paper are summarised in Table 1. This mixed methods study comprised: 1) mapping N50k programme objectives, proposed interventions and activities; 2) national survey of NHS trusts to determine the local impact of N50k; 3) analysis of data from national sources to determine national changes in registered nurse recruitment and retention; 4) case studies of two NHS trusts and their associated pipeline higher education institutions to provide in-depth insight into what has been done and how implementation varied. In the wider evaluation, we also undertook a cross-sectional survey of registered nurses’ experiences, and discrete choice experiment surveys of early-career and late-career job choices (not reported in this paper).

**Table 1:** Summary of study objectives and methods.

Objectives	Methods
Map proposed interventions and activity related to the N50k programme	Semi-structured interviews with programme leads and implementers; review of policy documents and internal reports
Determine local and national changes in recruitment and nurse retention	National survey of NHS trusts; analysis of routine data; case studies
Identify the impact of the programme from employer, university and nurse perspectives	National survey of NHS trusts; case studies
Evaluate how the programme worked in a range of contexts; in-depth insight into *what* has been done and *how* implementation varied.	Case studies

### 2.2 Ethical considerations

Ethical approval was obtained through University of Southampton Ethics and Research Governance Online for the overall study (ERGO 77532), NHS trust survey (ERGO 90253) and case studies (ERGO 98083). Health Research Authority approval was also obtained for the case studies (24/HRA/4128). Case study participants provided written informed consent before participation. The study was not registered.

### 2.3 Programme mapping of objectives and planned activities

Programme mapping was undertaken to understand the concepts underpinning the programme and describe the changes expected for the three workstreams (Table 2; supplementary tables S1-S3). Semi-structured individual or group interviews were conducted between March and September 2023 with 30 participants (selected for their involvement in the N50k programme delivery). Participants were national programme leads, regional and local implementers, and higher education leads. Interviews were recorded and transcripts generated. We did not undertake detailed thematic coding. Instead, key information from interviews was extracted and summarised alongside a review of 18 internal documents (logic models, Project Initiation Documents, programme overview reports). Using an Excel spreadsheet we recorded the objectives, scope, governance, planned activities, milestones and intended outcomes of individual programme strands. Nine programmes strands were outlined: seven domestic supply (undergraduate, postgraduate, pre-registration attrition, apprenticeships, conversion of nursing associates to registered nurses, nursing associate pipeline, return to practice), domestic retention, and international recruitment.

**Table 2:** Mapping of N50k programme objectives and key policy bundles / planned activities.

N50k programme objectives	Area of activity and examples of key activities
Domestic supply	
Increase undergraduate numbers by additional 3700 by March 2024 and 2400 full-time equivalent postgraduate numbers by March 2025	• Increase course enrolment via national campaign • Increase and monitor clinical placement capacity • Increase and support higher education capacity • Improve student financial support (learning support fund 5–8k) • Improve course flexibility e.g., hybrid learning
Reduce pre-registration attrition from 11.0% to 7.7% by 2024 (optimistic target between 1592 - 2955 full-time equivalent nurses*)*	• Improve measurement of course attrition • Improve financial support e.g., streamline payment processes • Improve sharing best practice across higher education • Improve clinical placement experience • Improve NHS transition e.g., career information
Increase apprenticeships by additional 1400 full-time equivalent by December 2025	• Improve NHS, higher education, Nursing and Midwifery Council readiness • Improve school and college campaigns • Increase career support to healthcare support workers • Increase nurse associate to registered nurse conversion
Increase return to practice by 6000 Full-time equivalent by 2025	• Support temporary registrants to return to permanent register • Improve nationwide campaigns • Support Return-to-Practice course completion
International	
Recruit additional 18,400 internationally educated nurses by March 2024	• Improve marketing - NHS as ‘destination of choice’ • Offer financial incentives to trusts £10 million in 2020 • Improve / standardise experience e.g., pastoral support • Improve higher education collaboration / reduce competition • Improve regional teams support • Accelerate development support e.g., recognise experience • Pilot refugee nurse support • Improve entry processes e.g., increase Objective Structured Clinical Examination capacity; mitigate visa challenges • Ensure trust ethical recruitment • Government-to-government agreements with more countries
Retention	
N50k optimistic target: reduce domestic leaver rate from 6.28% to 5.63% and international leaver rate from 9.2% to 7.2% (yielding estimated 7308–7508 full-time equivalent nurses (4478 domestic; 2620 non-UK)	• Analysis to target interventions at those most at risk of leaving • Support new starters - regional leads; legacy mentors • Support flexible working e.g., best rostering practice • Improve health and wellbeing support - e.g., self-help apps, expand occupational health; improve e.g. workload • Improve inclusive / compassionate culture - update of national freedom to speak up and whistleblowing policies • Pay and reward - top of band retention payments; review retire and return pension rules; convert bank into substantive roles • Increase learning and development for leaders/managers; set out guidance for leaders to foster compassionate leadership • Equality, diversity and inclusivity - Review COVID-19 impact on vulnerable staff groups; develop specific support for ethnic minority colleagues; good practice workshops

### 2.4 National survey of NHS trusts

A national online survey to describe recruitment and retention activity of nurses was sent to the Director of Nursing (or deputy) and the Human Resources Director at all 210 English NHS trusts (n = 420) in February 2024. The survey included categorical and open questions about N50k interventions, views about their impact, and about what improvements could be made. To achieve a response from at least 50 % of trusts, four e-mail reminders were sent, and non-response was followed up by telephone. The survey remained open until November 2024. The analysis presents descriptive statistics (numbers, percentages). For ease of presentation, 5-point Likert scale variables (e.g., strongly agree to strongly disagree) are combined into a three-category variable (i.e. agree, neither agree nor disagree, disagree).

### 2.5 Data analysis of the domestic pipeline

Data from Higher Education Statistics Agency provided the number of new entrants to nursing higher education courses in the UK. University and Colleges Admissions Service data provided the number of applications to undergraduate nursing degree courses. These were analysed to observe trends in the student pipeline between 2014 and 2024. The descriptive analysis presents the number of entrants to nursing higher education courses, applicants to nursing courses, students enrolled on courses, and numbers of qualifiers. Data for postgraduate qualifiers were not available separately to pre-registration students.

### 2.6 Data analysis of NHS joiners and leavers

Monthly employment Human Resources and payroll data of approximately 31 million monthly staff from NHS Electronic Staff Records, was used to identify factors associated with nurses leaving NHS hospital and community services in England. Eligible registered nurses were identified using pay band data. Pay bands are determined by the NHS Agenda for Change, based on knowledge, skills, and responsibilities (NHS Employers, 2026). Band 5 broadly represents registered staff nurses delivering and coordinating direct care and is the starting point for qualified nurses. Band 6 typically includes experienced nurses in specialist, senior staff nurse or team-lead roles. Band 7 typically includes advanced specialist nurses, team leaders and ward managers with greater clinical, leadership and managerial responsibility. Bands 8b–9 are senior professional, service or organisational leadership roles. Nursing assistants and associates were excluded (below pay band 5) from the analysis. Pay band was treated as a baseline characteristic and was used for subgroup analysis.

We examined the association between leaver rates and demographic characteristics, nationality group, employment characteristics, pay band, nurse wellbeing (sickness absence for physical and mental health conditions), and local area factors (local wider labour market measures). Nationality was derived from the Electronic Staff Record data and grouped into three mutually exclusive categories: UK; EU/EEA (all recorded EU/EEA nationalities other than UK); and non-UK/non-EU/EEA (all other recorded nationalities) labelled ‘rest of the world’ in the analysis. These categories indicate recorded nationality, not country of birth, country of nurse education or route of recruitment. The ‘rest of the world’ category is heterogeneous, combining nationalities from higher-income countries (with health systems broadly comparable to the UK, such as Australia, New Zealand and Canada), with those from countries that differ in health-system, educational and economic context. This aggregate category was used because available data did not support sufficiently robust country-level analyses.

We compared two broad periods of time: January 2020 to June 2023 (N50k period) with January 2016 and June 2019 (pre-N50k). We described joiner rates by reported sources of recruitment, demographic characteristics, and the trend over time. We analysed monthly employment data for any individual with a nursing role in hospital and community service providers (NHS trusts) and NHS arm’s length bodies in England for at least one month during the period April 2015 to June 2023. We used multiple regression to analyse the *risk of leaving* based on the Cox proportional hazards model. Time-to-event was measured from NHS entry date (regardless of whether individuals joined the NHS in a non-nursing role) until the point they left their registered nursing post. If they did not leave by the end of the study period, these records were censored. Analysis was conducted using the PHREG procedure in SAS software. We ran models to explore categories of factors potentially associated with leaving: 1. temporal (year, month); 2. demographic (age, ethnicity, nationality, gender); 3. employment (pay band, pay point, sickness absence). A hazard ratio (HR) of >1 indicates higher likelihood (risk) of leaving and <1 indicates a lower risk (HR = 1 no difference). If the Confidence Interval (CI) excludes 1, the result is statistically significant. Adjusted hazard ratios (adjusted by all variables reported on) are also presented.

### 2.7 Case studies

We aimed to select three contrasting case study sites to provide an in-depth insight into what has been done and how implementation varied in different organisational contexts. We initially identified nine potential cases selected for their geographical location, local population characteristics, level of N50k activity determined from the NHS trust survey (e.g., interventions, activities, perceptions of programme impact) and changes in nurse recruitment/retention (number of apprenticeships, joiner/leaver rates, proportion of international nurses). Of nine, three agreed to participate but we could only collect data in two sites (as the third site was unable to deliver participant recruitment).

Each case study comprised an NHS trust and local higher education institutions. Case 1 comprised a large acute teaching hospital in the south of England with a catchment area of approximately two million patients, employing over 4000 nurses. It also included five local universities. Case 2 comprised a large acute teaching hospital in the north of England with a catchment area of over 600,000 patients and employed approximately 4000 nurses. Case 2 included four local universities. Between January and May 2025, we conducted 31 semi-structured interviews (17 at Case 1; 14 at Case 2) with fifteen NHS trust leaders/managers nine education leaders from nine universities, and seven nurses and students across the two cases. The paper presents findings across the two case sites rather than presenting separate case studies but we draw out any case specific findings.

Topic guide questions were adapted to trust and education leaders, nurses and students (supplementary material). Video or face-to-face interviews (depending on participant preference) were undertaken by three research team members (JE, JF, JT) and lasted between 40–60 min. Interviews were audio recorded with consent, and transcribed verbatim. Framework analysis informed the approach (Ritchie and Spencer, 2002) and we combined deductive codes developed from a preceding literature review (Ejebu et al., 2024) and the NHS trust survey, with additional inductive coding of data. Early transcripts were independently coded by JF, JE, and JT. Emerging codes were discussed as a team and incorporated into a refined framework with definitions of codes, which was applied across both cases. Data were charted into matrices to support within- and cross-case comparison. We developed themes and narrative summaries through iterative interpretation and consensus. NVivo 15 (Lumivero) was used to manage and retrieve data.

## 3 Results

### 3.1 Overall assessment of the N50k programme

Table 2 shows overview of the aims of the N50k programme, key interventions and areas of key activity, derived from the internal programme documents. Further details are reported in supplementary tables (S1 domestic, S2 international and S3 retention).

Case study findings and 162 respondents to the NHS trust survey (from 119 different trusts) showed the N50k programme enabled new activity and accelerated substantial structural and operational changes - notably, rapid expansion of international recruitment, and some support of apprenticeships. Respondents to the open questions on the survey, and case study participants, were particularly concerned about a lack of connectivity between the three workstreams and differential investment across streams (e.g., nurse degree apprenticeships; international recruitment). The cyclical nature of national and local level health funding, and target-driven gains were perceived as fragile and characterised as repeated expansion and contraction that is detrimental to longer term workforce planning:

The only way you were going to get [50,000] was … with international recruitment. ‘Grow your own’ [e.g., apprenticeships, undergraduate] … it’s not a standing start (Trust leader 1, Case 1).

We met the numbers in May 2024 … but we’ll just watch them go again on a tide (Trust leader 3, Case 1).

### 3.2 Increasing domestic supply

The domestic supply workstream included discrete strands of activity, set out in the Project Initiation Documents (Table 1). There was a particular focus in the documents on increasing undergraduate, postgraduate and apprenticeship numbers.

*Increasing undergraduate expansion:* Whilst the number of new entrants was higher in 2023/24 compared to 2019/20 (Fig. 1), the overall trend since 2015 was downwards, suggesting that N50k interventions may have stabilised rather than increased student numbers. There were 26,990 undergraduate qualifiers at UK higher education institutions in 2019/20 compared to 30,695 in 2023/24, an increase of 3705, delivering the target of an additional 3700 full-time equivalent registered nurses. Further, Universities and Colleges Admissions Service applications have declined year on year following a COVID-19 peak in 2021 (approximately 33,000 in 2019 compared to 31,000 in 2024).

**Fig. 1:**
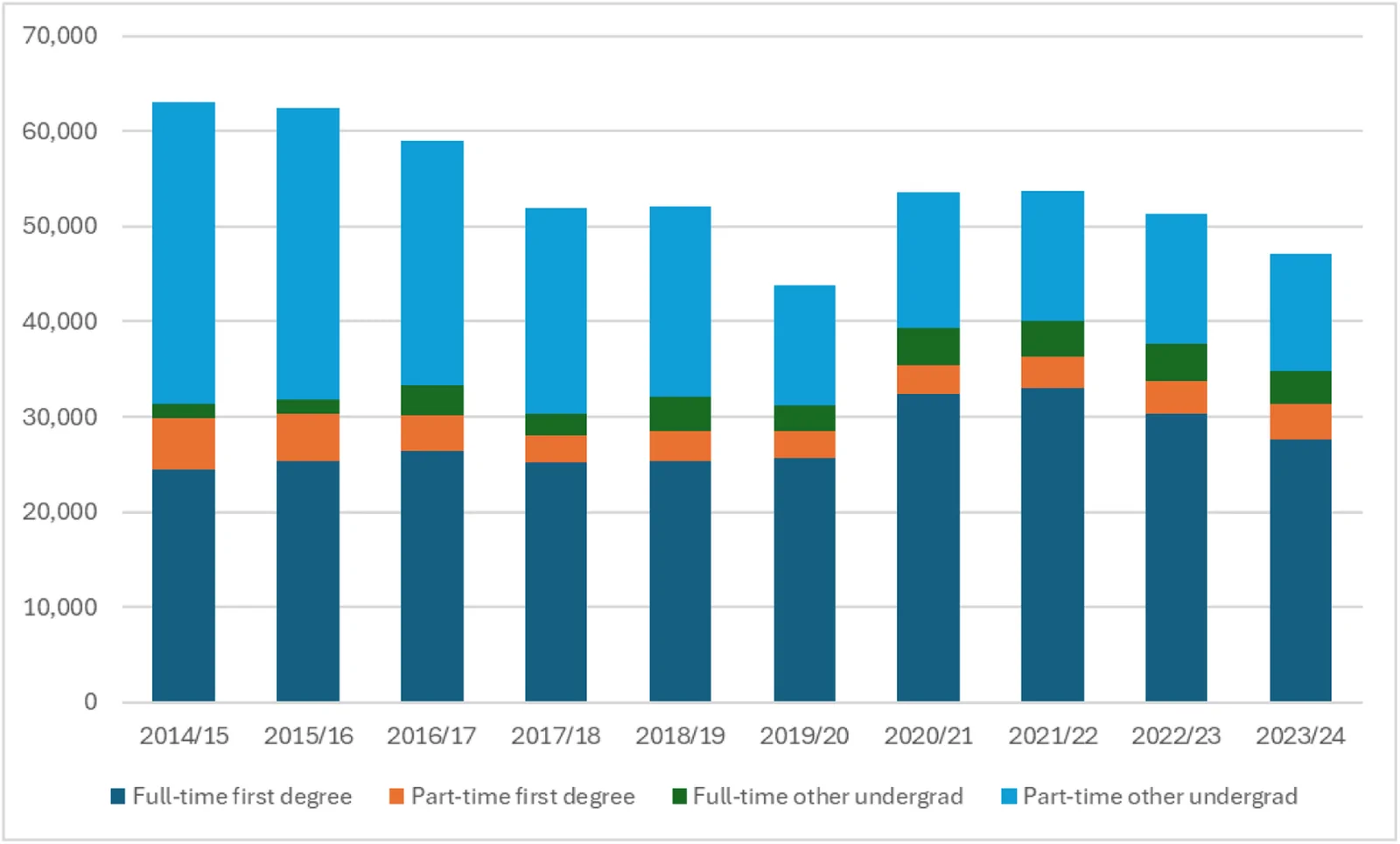
Number of new entrants to nursing courses at UK higher education institutions between 2014/15 to 2023/24. Source: HESA Student data Table 52. ‘First degree’ refers to a Batchelors degree enabling registered nurse status. ‘Other undergraduate’ includes courses excluding bachelor’s degrees (courses with qualification aims equivalent to and below first degree level e.g., foundation degrees, Higher National Diploma).

Of 157 trust survey respondents (five missing responses), only 57 (36.3 %) perceived that N50k would continue to support the domestic workforce supply beyond 2024. Of 135 trust survey responses to an open text question that asked, ‘what would most improve domestic supply’, 54 cited apprenticeship funding and backfill of salaries, 25 student bursary and financial support, 14 pay and conditions, and 13 cited recruitment and wider participation, with smaller numbers naming flexible working, progression and recognition, and increased placement capacity. Investment in apprenticeships was widely supported in trusts, but these required greater funding and salary backfill. Whilst the loss of bursaries was partially offset by the more modest Learning Support Fund in 2019 (non-repayable supplementary financial support, including an annual training grant and support with placement-related travel, accommodation, and childcare), lack of financial support was seen as harmful by survey respondents, particularly in attracting mature students. Case study participants’ views largely reflected those of trust survey respondents. Out-of-pocket student expenses on clinical placements (e.g., travel costs) and slow reimbursement were potential barriers, alongside a perceived insufficient Learning Support Fund.

The fees issue is fundamental … the NHS does pay something … but [students also] need a student loan … you're asking people to come out with 40, 50,000 pounds of debt (Education lead 3, Case study 1).

Participants in both case sites emphasised the need to better portray nursing as a skilled, autonomous profession. National advertising campaigns were a key N50k intervention, but these were not visible to many participants:

To turn around the decline … is going to take a national coordinated and funded campaign (Education lead 4, Case 1).

We see on the television recruitment campaigns for teachers … we don’t understand why there hasn’t been one for nursing (Education lead 1, Case 2).

Universities have diversified courses to attract and accommodate candidates where flexibility is key, offering multiple entry routes, and increasing accessibility, alternative start dates, smaller cohorts, or creating shorter pipelines into registration. However, education leaders viewed these as not boosting overall student numbers but rather enhancing access and inclusivity:

It’s more inclusive because it doesn’t rule out people who perhaps haven’t got the Further Education qualifications (Education lead 3, Case 2).

The N50k objective to increase and support university capacity is difficult to assess. Education leaders from both case sites reported a lack of visibility, ambiguity and uncertainty about N50k resources and activities. Increased collaboration via regional consortiums of higher education institutions and NHS trusts was reported. However, education and some trust participants were concerned about a lack of involvement in national level decision-making.

Some simulated practice money might have come that way, but nothing has drawn my attention to this as part of N50k (Education lead 3, Case 1).

We have to work in partnership … it’s critical to have a seat at that table but we don’t seem to be getting the invites (Education lead 2, Case 2).

Programme mapping indicated that initiatives to increase NHS clinical placements was supported by substantial funding. Of 156 trust survey responses (6 missing), 74 (47.4 %) agreed that placement capacity had increased due to N50k funding, whilst 39 (35.0 %) were neutral and 43 (27.6 %) disagreed. Some case site education leaders perceived placement shortages existed, with one reporting workarounds, for example, introducing placements in the university setting.

We had to have a corporate placement within the university because there wasn’t enough placements (Education lead 3, Case 2).

They perceived placement shortages particularly in mental health and the private/voluntary sector. Education leaders perceived a lack of coordination across NHS trusts in relation to placements. In contrast, trust participants typically cited underused placements, but some acknowledged that ensuring sufficient and willing supervision could be challenging.

Our biggest mental health trust can’t even give our student nurses placements (Education leader 1, Case 2).

Not everybody [in the workforce] quite gets that every single nurse is a practice supervisor … which is really disappointing (Trust leader 3, Case 1).

Some case study participants suggested there was greater use of simulation-based learning as part of nursing education, but others reported these had cost and space constraints. However, one trust participant perceived simulated learning was unnecessary, and inadequate.

It’s not clinical and the nursing students need that clinical … experience. (Trust leader 4, Case 2)

Simulation hasn’t been picked up by universities … it’s cheaper to send them out (Trust leader 3, Case 1).

*Increasing apprenticeships and nurse associate pipeline:* Apprenticeship enrolments increased and the N50k numerical target was met, but expansion depended heavily on salary-backfill funding and was unlikely to be sustained once programme support ended. Financial support of £172 million for apprenticeships and £8300 to trusts per apprentice per year aimed to increase numbers by an additional 1400 full-time equivalent nurses (Table 2). Between 2020–21 and 2023–24 annual enrolments on registered nurse degree apprenticeships were 2200 in the academic year 2020/21 and 3400 in 2023/24 (Department for Education, 2024), compared with 938 in 2019/20, indicating that the N50k target was met. During the N50k programme, focus shifted to apprentices on shorter programmes (e.g., two-year nurse associate and associate practitioners’ conversion to registered nurse programmes) to meet the N50k programme timeline. Of 157 survey respondents (five missing responses), 91 (58.0 %) agreed that apprenticeship intakes increased at their NHS trust between 2020–2024, and 109 (69.4 %) agreed that funding for staff salary backfill at their trust was critical to enable expansion. However, only 28 (17.8 %) agreed that ‘N50k contributed to apprenticeship growth’ and 38 (24.4 %) agreed that ‘N50k funding would continue to have a positive impact on apprenticeships beyond 2024’.

Given the challenges of attracting undergraduates, apprenticeships were viewed as a necessary strategy by survey respondents and case study participants. Since apprenticeships offer a salary, some may view these as more attractive than direct entry routes. NHS trust participants also perceived that apprenticeships aligned with organisational values:

People … parents have woken up to the apprenticeship alternative [] We embraced the apprenticeship … we were the first trust to work with [named university] (Trust leader 2, Case 1).

However, there were concerns about the financial challenges of providing apprenticeships. These programmes rely on a levy to fund training, but salary backfill costs are significant:

To backfill fifty nurses … for three days a week cost us 2.something mill [pounds] … that was helped by [N50k] … the apprenticeship levy won’t pay for any backfill, only training costs (Trust leader 1, Case 1)*.*

*Reducing nursing programme attrition:* No national level data was available, but qualitative data from the case studies suggested that earlier retention initiatives (Health Education England, 2018) and Schwartz rounds (Golding, 2024) were viewed as positively reducing attrition, particularly in addressing the “*year one to two wobble*” in undergraduate courses. Education leaders all reported they provided a high level of student support (e.g., pastoral tutoring) to ameliorate attrition. COVID-19 adaptations (e.g., use of videocalls for check-ins) led to hybrid models of support in some universities, which improved efficiency and accessibility when students were on placement:

We can now check-in on a student many more times … we just don’t have to spend two hours getting there (Education lead 4, Case 1).

Smaller cohorts and individualised support at universities enabled early intervention. This was particularly needed when students had poor placement experiences:

We’re able to identify early doors that there’s an issue … and offer support … once we’ve got them, they stay with us (Education lead, Case 2).

*Increasing the number of nurses returning to practice in the NHS:* The N50k programme had an ambitious target for contributing 6000 additional full-time equivalent nurses back to the NHS (Table 2). COVID-19 interrupted activity since most Return-to-Practice courses and clinical placements were suspended. While there was no data on the net impact, NHS workforce data indicates that 4600 of 10,300 (44.0 %) registered nurses who retired between July 2021 and June 2022 rejoined the health service within 12 months, a 4.0 % increase in returning numbers from the previous year (Smail, 2023). The increase is partly attributed to an extension to wider pension rule changes in 2020, so that certain health workers retiring at 55 years can return to the NHS without impact on their pension, even when working full time. Some case study participants viewed N50k return on investment as low, but recognised the contribution of these staff, and returners were supported.

Always been a really, really low return… We’ve got two this year, I think… most were wanting to come back to … something less acute. It’s not a big stream but we still support it (Trust leader 1, Case 1).

### 3.3 Increasing international recruitment

International recruitment was the principal driver of growth, substantially exceeding the N50k target, but its long-term contribution depends on sustained investment in onboarding, integration, career progression and retention. Table 2 shows key areas of activity that were intended to increase and support international recruitment and recruit an additional 18,400 internationally educated nurses by March 2024. Between January 2020 and June 2023, 92,714 nurses were new (i.e. additional) to the NHS workforce (‘joiners’). Of these, 40,531 were from outside of the UK, EU/EEA countries (‘rest of the world’) accounting for 43.7 % of additional nurses, over double the N50k target of 18,400. This compares to 9066 nurses from ‘the rest of the world’ countries joining between January 2016 and June 2019, a 347 % increase. Nurses from EU/EE Area countries were at higher risk of leaving relative to nurses of UK nationality (HR 1.49, 95 % CI 1.47 - 1.52). Nurses from all other ‘rest of the world’ countries had a lower risk of leaving (HR 0.62, CI 0.61 - 0.63). Nurses from the ‘rest of the world’ had the lowest leaver rates (adjusted for other demographic and employment factors) in 2020/21 (HR 0.64, CI 0.60 - 0.67). However, findings suggest that leaver rates increased in this group (HR 0.82, CI 0.80 - 0.85) in 2023/24 but were still lower than UK-nurses. Case study participants reported that some nurses may leave for other countries (e.g., Australia, USA) after three years, making long-term retention an ongoing concern.

*Improving infrastructure and regulatory processes:* N50k funding enabled international recruitment at scale. Trust survey respondents and case study participants typically described recruitment as resource-intensive but an essential stream of workforce expansion. Of 138 trust survey respondents to an open question about challenges of international recruitment experienced, 25 cited the cost of living and high accommodation costs for newly arrived nurses, 46 cited recruitment, regulatory and onboarding challenges (visa and registration delays, training capacity, cultural transition). A further 20 respondents cited a lack of support (e.g., inadequate pastoral support, preceptorship, supervision).

Whilst substantial N50k funding was made available, some case study participants reported that it did not cover the full costs, for example, accommodation, pastoral support and Objective Structured Clinical Examination (OSCE) training (an assessment method for evaluating clinical skills used in many healthcare contexts). This created some planning instability, and cost burdens were sometimes perceived as unfair. Being part of a regional collaboration of NHS trusts allowed some cost-sharing of recruitment agency fees, OSCE training for neighbouring trusts and coordination costs (e.g., airport pickups) which alleviated some of the shortfall.

The advantage of N50k was there was some funding, but it wasn’t adequate … average [recruitment cost] is about nine and a half to 11,000 pounds depending on which country we are recruiting from … the average tariff is about five thousand pounds … the trust has to fund that gap (Trust leader 1, Case 2).

Some organisations are doing it on their own, but we are lucky because we were part of the [regional] consortium (Trust leader 4, Case 2).

Historically, both case study trusts had experience of international recruitment, but N50k funding enabled rapid expansion and a more structured approach.

[It improved] pastoral support … and supervision. It enabled us to get some premises [to] focus specifically on delivery of education [] People in the HR team … helped people … to settle into accommodation (Trust leader 1, Case 1).

*Improving early support facilitating readiness and confidence:* At both case study sites, trust-led OSCE training was seen as leading to better outcomes compared to external training. Trust leaders and internationally educated nurses themselves emphasised the importance of practical and pastoral pre-arrival and on-arrival support, as well as integration into roles. Support included airport pickups, OSCE training, and cultural peer support. Both case sites reported that funding supported improving induction, training and preceptorship, and this was well received by nurses.

Trained by our education team, being prepared for the OSCE … they give us that intensive training that was really helpful (Nurse 6, Case 1).

In one case study, some participants recognised that OSCEs placed substantial pressure on newly arrived international nurses and the trust provided a high level of support. However, some felt there may not be enough focus on training on NHS protocols and local policies. OSCE preparation was perceived as more difficult for some cohorts than others, attributed to differences in clinical experience, culture or language skills. Some felt that more tailored approaches may help accommodate this diversity.

If you don’t pass your OSCE in three go’s, you go home [] There’s so much bound up in the OSCE it’s hard for them to … really settle into their clinical area and start to develop (Trust leader 5, Case 1).

Often, they’ve got the clinical skills and knowledge. It’s the wider stuff around the cultural piece and interactions with patients, with colleagues, they need more support with (Trust leader 2, Case 1).

They don’t want to make a risky move because they have to battle so much for their PIN [registration] (Nurse 3, Case 1).

While standardising OSCE training is necessary for patient safety and regulation, findings suggest that more tailored preparation may better support some cohorts. The need for preceptorship and pastoral support is crucial, but may need to start earlier.

The preceptorship programme … should start as soon as you go to the ward [] I had it later after OSCE … but already we were in the wards … it comes in [at] three months (Nurse 7, Case 1).

Case site 2 portrayed a strong focus on creating belonging through peer-led support (e.g., forums, events, signposting), training, inclusive leadership efforts, and pastoral care. Previous experience of employing cohorts of international nurses (particularly Filipino nurses) helped ease transitions for newcomers. The trust offered formal and informal support e.g., 24/7 hotlines, WhatsApp and Facebook groups, cultural celebration events, and support with local language.

[Newly arrived nurses] do brilliantly … there’s a ‘school’ that is put on by the hospital called: ‘the [accent] school’. They learn … about the [city] culture … really well attended … they think it’s hilarious (Trust leader 5, Case 2).

*Career opportunities for international nurses:* Some trust participants perceived a growing dependency on international nurses, particularly for hospital-based ward care. It is important for equality – and retention – that internationally-educated nurses can access opportunities across the NHS. Ward roles may be seen as a stepping stone to other specialist or community roles, thus creating opportunities are important. Key retention factors for international nurses included career development support and confidence building e.g., peer support networks, mock interviews. Some participants noted a lack of diversity in leadership, sometimes attributed to the relatively recent arrival of larger numbers of overseas nurses. However, there was also some awareness of potential cultural and internal progression barriers.

We’ve got a better number of international nurses in more senior posts. What we don’t do well is encourage these nurses to come into specialist roles [] We’ve got some fantastic nurses … [but] they go: ‘not yet, maybe I’m not ready’ (Trust leader 5, Case 2).

[Some] still find English as a barrier in communicating with patients … they are very much aware that if that is a struggle how will they manage and lead a team? Confidence is a big thing in leadership (Nurse 3, Case 1).

### 3.4 Retention

Nurse retention improved during the N50k period, but the programme’s direct contribution was unclear. Sustained retention depended on local workplace culture, supportive teams, flexibility and career development. The leaver rate reduced from 6.28 % to 5.63 % for nurses trained in the UK (domestic) and from 9.2 % to 7.2 % for nurses trained overseas over the N50k period. Programme documents indicated that efforts focused on improving flexible working practices, health and wellbeing support, and the workplace environment. Increasing pay was not an N50k intervention (Table 2). Of 140 survey respondents 116 (82.8 %) agreed that retention rates had improved at their trust since 2019. However, only 42 (30.0 %) agreed ‘N50k had a significant effect on their trust’s ability to retain nurses’, and 51 (36.4 %) agreed ‘N50k would have a positive retention effect after April 2024’ (i.e., beyond the end of the programme).

Registered nurse retention rates were already improving prior to the N50k programme, but they were subsequently impacted by COVID-19. The lowest rate of leaving was in 2020/21 (including when adjusted for all demographic and employment factors (HR 0.78, CI 0.76 - 0.79 compared to 2015/16)). This was followed by two years with leaver rates above the pre-pandemic (2019/20) level which largely reflects some nurses postponing leaving in the first year of COVID-19 (2020/21) until subsequent years. By 2023/24 leaver rates were similar to the pre-pandemic year (Table 3). Broadly, likelihood of leaving increases with each age band category, from those under 25 years (HR 0.91, CI 0.89 - 0.93, compared 25–34 year olds) up to those aged over 65 (HR 3.86, CI 3.75 - 3.97). However, the 35–44 year age category does not fit that trend as leaver rates amongst that cohort (HR 1.07, CI 1.06 - 1.08) were higher than for the immediately older age category (HR 1.00, CI 0.99 - 1.02). Male nurses were significantly more likely to leave compared to females, even after adjustment for demographic and employment characteristics (HR 1.23, CI 1.22 - 1.25).

**Table 3:** Risk of leaving (hazard ratio) by demographic and employment factors.

Variable		HR [CI](unadjusted)	HR [CI] (demographic and employment adjusted)*
Year	2015/16	ref	ref
	2016/17	1.02 [1.01,1.04]	1.01 [0.99,1.02]
	2017/18	1.01 [0.99,1.02]	0.99 [0.97,1.01]
	2018/19	0.97 [0.96,0.99]	0.96 [0.94,0.98]
	2019/20	0.89 [0.88,0.91]	0.88 [0.87,0.90]
	2020/21	0.81 [0.80,0.83]	0.78 [0.76,0.79]
	2021/22	1.04 [1.02,1.05]	1.00 [0.99,1.02]
	2022/23	0.97 [0.95,0.98]	0.95 [0.94,0.97]
	2023/24	0.83 [0.80,0.85]	0.88 [0.86,0.91]
Nationality group	United Kingdom	ref	ref
	European Union/European Economic Area	1.49 [1.47,1.52]	1.40 [1.38,1.43]
	Rest of World	0.62 [0.61,0.63]	0.78 [0.77,0.80]
Gender	Female	ref	ref
	Male	1.23 [1.21,1.24]	1.23 [1.22,1.25]
Age band	21–24	1.01 [0.99,1.03]	0.91 [0.89,0.93]
	25–34	ref	ref
	35–44	1.09 [1.08,1.11]	1.07 [1.06,1.08]
	45–54	1.08 [1.06,1.10]	1.00 [0.99,1.02]
	55–64	2.07 [2.04,2.10]	1.78 [1.75,1.81]
	65+	4.65 [4.52,4.77]	3.86 [3.75,3.97]
Agenda for Change Payband	Band 5	ref	ref
	Band 6	0.82 [0.82,0.83]	0.73 [0.72,0.74]
	Band 7	0.81 [0.80,0.83]	0.73 [0.72,0.74]
	Band 8 - Range A	1.02 [1.00,1.04]	0.93 [0.91,0.95]
	Band 8 - Range B	1.24 [1.19,1.28]	1.10 [1.05,1.15]
	Band 8 - Range C	1.41 [1.34,1.49]	1.26 [1.19,1.34]
	Band 8 - Range D	1.55 [1.43,1.68]	1.36 [1.24,1.49]
	Band 9	1.62 [1.43,1.83]	1.26 [1.08,1.46]
Spine point level	Bottom	ref	ref
	Middle	0.97 [0.96,0.98]	1.00 [0.99,1.01]
	Top	1.02 [1.01,1.04]	0.93 [0.92,0.95]

*Results were adjusted to account for demographic and employment differences between groups.

There is a U-shaped relationship between pay band and leaver rates, with Band 6 and 7 nurses less likely to leave relative to Band 5, after adjusting for demographic and employment factors (HR 0.73, CI 0.72 - 0.74; HR 0.73, CI 0.72 - 0.74). Bands 8b-9 were more likely to leave, which might be explained by those in higher bands moving to non-clinical management roles. Those at the top of pay bands were (after adjustment) overall less likely to leave (HR 0.94, CI 0.93 - 0.95).

Sickness absence is a significant predictor for three categories of absence (mental health; flu, cough, and infectious disease; other conditions) relative to those with no sustained absences. The risk of leaving increases with increasing number of sick days taken. In particular, nurses with >84 days of mental health related absence in a three month period had over 6.5 times the risk of leaving their role compared to those with 0–4 days of mental health sickness absence (Table 4).

**Table 4:** Risk of leaving by type of sickness absence (number of days absent in a three-month period).

Variable	Number of days absent in 3 month period	Hazard ratio [CI](unadjusted)	Hazard ratio [CI] (demographic and employment adjusted)
Mental health	0–4 days	ref	ref
	5–14 days	2.05 [1.99,2.12]	2.16 [2.09,2.23]
	15–24 days	2.26 [2.18,2.34]	2.38 [2.29,2.48]
	25–44 days	2.48 [2.40,2.56]	2.67 [2.58,2.77]
	45–64 days	2.98 [2.87,3.11]	3.22 [3.08,3.36]
	65–84 days	3.47 [3.30,3.65]	3.86 [3.66,4.07]
	85+ days	6.30 [6.11,6.49]	6.74 [6.51,6.98]
Flu, cough, infectious disease	0–4 days	ref	ref
	5–14 days	1.04 [1.02,1.06]	1.19 [1.16,1.21]
	15–24 days	1.15 [1.09,1.20]	1.27 [1.20,1.34]
	25–44 days	1.42 [1.32,1.52]	1.54 [1.49,1.59]
	45–64 days	1.74 [1.55,1.95]	1.83 [1.61,2.08]
	65–84 days	1.94 [1.66,2.27]	2.01 [1.69,2.40]
	85+ days	2.77 [2.53,3.03]	2.83 [2.56,3.13]
All other reasons	0–4 days	ref	ref
	5–14 days	1.32 [1.30,1.34]	1.39 [1.36,1.41]
	15–24 days	1.34 [1.30,1.38]	1.39 [1.35,1.44]
	25–44 days	1.49 [1.45,1.54]	1.54 [1.49,1.59]
	45–64 days	1.83 [1.76,1.90]	1.88 [1.81,1.96]
	65–84 days	2.09 [1.99,2.19]	2.21 [2.10,2.33]
	85+ days	4.68 [4.56,4.80]	4.60 [4.46,4.73]

* Results were adjusted to account for demographic and employment differences between groups.

*Retention and sustainability:* Case study participants were uncertain if direct N50k funding was available to support retention compared to recruitment activity. Investment and initiatives were provided at the regional level via ‘retention hubs’, but these were characterised as remote. Local organisational factors (e.g., ward culture) were cited as more important than top-down schemes.

This is about retention and recruitment. There’s no point filling a bucket with a hole in it … I’ve seen a lot of N50k projects … the sustainability bit is lost [] I think there was [N50k] money. Not for us directly but … regionally … which have now stopped (Trust leader 1, Case 1).

It’s the bit on the ground that makes the difference whether your nurse will want to stay on that ward, or trust, not necessarily the stuff around the region [] The reason you come into work … is the team you work with (Trust leader 4, Case 1).

Participants cited the importance of preceptorship improvements, wellbeing initiatives, Professional Nurse Advocate roles (which aim to improve staff wellbeing, retention, and patient care through clinical supervision and support) and legacy mentors (experienced nurses providing coaching, and pastoral support to newly qualified or newly arrived nurses)*.* COVID-19 crisis management facilitated some positive changes.

COVID survival was about helping those staff cope, to work another day and to stay another year, and to stay another five … all of the things around wellbeing, professional nurse advocates (Trust leader 1, Case 1).

In Case 2, low vacancy levels were attributed to proactive vacancy management but also to an attractive workplace, e.g., access to parking, accessible location, supportive leadership and education culture. Participants cited positive turnover and opportunities elsewhere rather than push factors, reflecting a healthy culture of development and mobility. Supporting the transition from qualification to post-registration was recognised as a critical phase in both case sites, citing preceptorship, legacy mentoring and early-career support structures, to prevent early exiting, and key to long-term workforce stability.

We’ve got a very good preceptorship programme … they tend to leave because they’ve got an opportunity rather [than] they don’t like us (Trust leader 2, Case 1).

Professional nurse advocacy, coaching, supervision, working alongside [experienced colleagues] … first, two, year support is really important (Trust leader 1, Case 2).

Participants from both case sites focused on the importance of relational leadership and ‘knowing your staff’ for contributing to staff wellbeing and staff feeling valued. Wider support included menopause support and financial wellbeing (e.g., pension advice, bank sessions). Rather than lose staff due to workplace dissatisfaction, both trusts used internal transfers or intervened where possible.

Where there’s been a [clinical] team that’s not working … we have gone in to try and address those things … improve the working environment (Trust leader 2, Case 1).

We introduced an ‘itchy feet’ process … if I had a vacancy and somebody in another area said: “look, I’m having a really hard time” … You transfer people in and out … without having to go through the whole recruitment process … Not massive numbers … but we stopped quite a few leaving (Trust leader 5, Case 2).

*Flexibility, facilities and demonstrating staff are valued:* Many trust and education participants in both cases perceived that career aspirations were now more dynamic, with younger nurses particularly seeking greater flexibility and portfolio careers challenging the ‘job-for-life’ model. Flexibility was also viewed as a practical and symbolic tool to demonstrate staff are valued. However, poor facilities led to some nurses feeling unvalued, as well as creating practical barriers to staying.

The younger generation … want to have much more of a work/life balance than [us] … flexibility is important if you are able to accommodate it … they value the organisation because it values them (Trust leader 2, Case 1).

[It] sounds a bit daft, but someone is leaving the emergency department because their car parking space … they've been told that they can't have it anymore. (Student 1, Case 1).

*Career development and opportunities:* Supporting career development was perceived as key to retention and attractive to nurses. Trust leaders at both case sites were less certain whether career support was directly attributable to N50k funding. Opportunities and support were reported as available, but it may rely on individual motivation to seek these out.

There is a whole pile of leadership stuff [] What I’m not sure is whether [N50k] gave us the [funded] CPD [Continuous Professional Development] … if it did, the upside was there’s always money for every nurse to go and do some CPD (Trust leader 2, Case 1).

There’s so many openings … [After] finishing my preceptorship programme, I will start looking into where I want to be, say, the next three years [] You can check on StaffNet [staff intranet]. There are career progression opportunities if we, as staff, look into it. You just need to check (Nurse 7, Case 1).

Some nurses had concerns about opportunities to progress from Band 5 to Band 6 since many Band 6 posts were filled via internal promotion. This suggested good internal career progression pathways but may be a barrier to moving across roles and organisations.

My friends applied for maybe 20 or 30 Band 6 roles … If you want to be a clinical nurse specialist, it's harder to get funding so it would have to be self-funded (Nurse 1, Case 1).

## 4 Discussion

*Has the programme been a success?* The N50k programme partly achieved its objectives. The Department of Health and Social Care reported 51,245 additional nurses in September 2023 compared with September 2019, meeting the manifesto commitment six months early (Department of Health and Social Care, 2023). In summary, growth was driven largely by international recruitment, which could expand faster than domestic supply. However, COVID-19 disruption and post-Brexit changes to EU/EEA recruitment and free movement (Dayan et al., 2025) intensified workforce pressures and complicated attribution of change to N50k.

N50k accelerated international recruitment, apprenticeships and regional collaboration, but pre-registration enrolments did not substantially increase. Student debt and placement costs, negative perceptions of nursing, limited campaign visibility and uncertain higher education involvement persisted. Flexible routes and increased placement capacity therefore appeared to stabilise declining enrolments rather than generate sustained growth.

Retention improved, but N50k’s contribution could not be separated from pandemic effects, wider policies and existing initiatives. Activity focused on flexible working, professional development and early-career support, while organisational culture and supportive teams appeared more influential. Success should therefore be judged not only by the headline target, but by whether gains in supply and retention are sustainable.

*What could be improved?* N50k demonstrates that a time-limited, politically driven target can rapidly increase nurse numbers, but may not necessarily establish a sufficiently strong domestic pipeline to sustain growth without continued international recruitment. The programme may be interpreted as an effective short-term expansion strategy rather than a conclusive solution to the underlying imbalance between workforce demand, domestic supply and retention.

The five-year N50k programme did not align with the time required to train sufficient numbers of undergraduate nurses. Future planning needs a coordinated trajectory of at least ten years, linking education commissioning, educator and placement capacity, student finance, course completion, transition into substantive posts and retention (Health Education England, 2018; NHS England, 2023; Palmer et al., 2023). It should also address stagnant applications, apprenticeship and backfill costs, attrition and graduate employment. Flexible routes may diversify access but appeared to stem decline rather than increase overall intake. Figures after the N50k programme suggest that undergraduate acceptances increased by only 1 % in 2025 (NHS England, 2025a). Closer coordination with higher education institutions and international recruitment plans is needed to avoid newly qualified nurses being unable to secure first posts (Royal College of Nursing, 2026).

Our findings support reducing students’ financial burden. External reports subsequently identified difficulty securing first posts (Pearce, 2025; Royal College of Nursing, 2026), prompting NHS England’s 2025 graduate guarantee, whose effect remains unclear (NHS England, 2025b). Apprenticeships widened access and were valued because they provided a salary and developed local staff. However, high backfill costs and depletion of the healthcare assistant workforce limit affordability without sustained investment. Future planning should compare apprenticeship expansion with stronger support for direct-entry students. Placement expansion also requires funded educator and supervision capacity. Potential reductions in placement hours and greater simulation use (Department of Health and Social Care, 2025b) should be aligned with accurate forecasting, particularly for neighbourhood and community care.

International recruitment enabled rapid growth and investment in training, onboarding and integration. OSCE preparation was extensive and well received, but support should better reflect varied experience, cultures and practice backgrounds. This accords with evidence that internationally recruited nurses require sustained integration, pastoral support, professional development and recognition of prior experience (Pressley et al., 2023). International recruitment was nevertheless the programme’s short-term success. Continued dependence creates exposure to global competition, migration policy and onward migration. Recruitment fell by 30.2 % between April 2024 and March 2025 (Nursing and Midwifery Council, 2025), alongside policy to reduce it below 10 % by 2035 (Department of Health and Social Care, 2025b). Without substantial domestic growth, rapid reduction risks recreating shortages. Targets should therefore specify applicants, entrants, qualifiers and NHS joiners over ten years, monitor losses throughout the pipeline and phase reductions against demonstrated domestic growth. International staff recruited during N50k also require sustained onboarding, inclusion and career progression, particularly as the UK may act as a stepping-stone to other English-speaking systems (Devereux, 2025).

Retention improved, but fewer than one-third of trust leaders attributed this directly to N50k. Although programme documents emphasised flexibility, ward culture, supportive leadership and teamwork were considered at least as important, consistent with wider evidence (Alkan et al., 2024; Conroy et al., 2023; NHS England, 2024). Career development received comparatively limited programme attention despite its importance for retention and NHS guidance (NHS England, 2021). Advanced practice, structured career conversations and flexible pathways may strengthen mid-career retention (Department of Health and Social Care, 2025b). Strategies should be sensitive to career stage and local labour-market context.

Overall, N50k was an effective short-term expansion strategy but did not resolve the imbalance between demand, domestic supply and retention. A sustainable workforce requires affordable entry routes, sufficient education and placement capacity, reduced attrition, funded transition into first posts and employment conditions that support retention. Targets should assess total growth, the balance of supply routes, entry into substantive employment, retention across career stages and whether gains persist after programme funding ends.

## 5 Limitations

This large-scale evaluation using multiple data sources is the first to report on a complex national policy intervention to expand a nursing workforce. The evaluation comprises complementary analysis of routine data and a national survey to provide a higher level, national view, combined with detailed qualitative data from case studies and open-ended survey questions. Together these are used to assess the success of this programme.

The domestic supply analysis is limited to published, aggregated data and is subject to limitations on reporting categories. The NHS leaver/joiner analysis is limited by some missing data (one in eight joiners had missing data). However, it reports trends over time, and it is unlikely these are data collection or classification artefacts. The broad ‘rest of the world’ nationality category may conceal important differences in retention between nationalities. Thus, hazard ratios should therefore be interpreted as average associations across a heterogeneous group rather than as applying uniformly to all constituent nationalities. From this evaluation using observational methods, it is not possible to isolate the impact of the N50k programme from other political and global factors (for example, COVID-19, Brexit, and wider healthcare retention policy initiatives). Two, rather than three, case studies were conducted, but combined with open-ended survey responses, these yielded rich qualitative data from two case sites and views from a large number of trusts. The evaluation did not include cost analysis, so it is not known if N50k investment is effective over the longer term. The evaluation includes self-reported views about impact, but we did not measure the effectiveness of specific initiatives (e.g., international onboarding and OSCE support). Further research is needed to understand longer-term impact on patient health outcomes.

## 6 Conclusions

The N50k programme succeeded in expanding the nursing workforce in England and met its headline target early. Growth was achieved predominantly through international recruitment rather than a sustained increase in domestic supply or a clearly attributable improvement in retention. The political commitment created targeted investment and focused action. However, much of this support was discontinued once the target was reached, placing gains in domestic training and retention at risk. Future workforce expansion therefore requires a long-term, coordinated strategy that strengthens domestic education routes, sustains effective retention measures and supports internationally recruited nurses.

## Ethics declaration

Written informed consent to take part in the study and to publish the article has been obtained from all participants or their legal representatives. The privacy rights of participants have been observed.

This study was performed in compliance with relevant laws, regulatory frameworks and guidelines where the research took place.

This study was approved by the through University of Southampton Ethics and Research Governance Online; Health Research Authority.

(Approval No ERGO 77532; ERGO 90253; ERGO 98083; 24/HRA/4128)

## Funding

This study/project is funded by the NIHR Policy Research Programme (NIHR203842). The views expressed are those of the author(s) and not necessarily those of the NIHR or the Department of Health and Social Care.

## CRediT authorship contribution statement

**Joanne Turnbull:** Writing – review & editing, Writing – original draft, Project administration, Methodology, Investigation, Formal analysis, Data curation, Conceptualization. **Lucina Rolewicz:** Writing – review & editing, Writing – original draft, Methodology, Investigation, Formal analysis. **William Palmer:** Writing – review & editing, Writing – original draft, Methodology, Investigation, Funding acquisition, Formal analysis, Conceptualization. **Julia Philippou:** Writing – review & editing, Methodology, Investigation, Formal analysis, Conceptualization. **Anne Marie Rafferty:** Writing – review & editing, Methodology, Investigation, Conceptualization. **Matthew Williams:** Writing – review & editing, Methodology, Funding acquisition, Formal analysis, Conceptualization. **Jane Prichard:** Writing – review & editing, Investigation, Funding acquisition, Conceptualization. **Ourega-Zoé Ejebu:** Writing – review & editing, Methodology, Formal analysis, Conceptualization. **Jane Frankland:** Writing – review & editing, Formal analysis, Data curation. **Jaimie Ellis:** Writing – review & editing, Project administration, Formal analysis, Data curation. **Iain Atherton:** Writing – review & editing, Methodology, Funding acquisition, Formal analysis, Conceptualization. **Michelle Jamieson:** Writing – review & editing, Methodology, Funding acquisition. **Jane Ball:** Writing – review & editing, Project administration, Methodology, Investigation, Funding acquisition, Formal analysis, Conceptualization.

## Declaration of competing interest

The authors declare that they have no known competing financial interests or personal relationships that could have appeared to influence the work reported in this paper.
