# Supplementary material for: Has a national programme to expand the nursing workforce in England been a success? A mixed methods study
Source: Int J Nurs Stud Adv. 2026 Nov 5;11:100735. doi: 10.1016/j.ijnsa.2026.100735 (PMC13770563; doi:10.1016/j.ijnsa.2026.100735)
Supplement: Supplementary file 1 [file mmc1.docx]

**Discussion Guide – Case studies**

**Nursing Workforce Leaders and Education Providers**

**About you and your role**

1. Can you tell me about your role,

- Length of time in post, length of time at this organization; leadership responsibilities, relationship to nursing workforce

Trust leaders

2. If in post pre-2019: Have you had any responsibility for the recruitment and retention of the nursing workforce more generally?  What were your responsibilities / involvement?

**N50K Programme**

3. Have you heard of the ‘N50K programme’?

Yes:

- What is your understanding of it? What are the programme’s objectives?

No:

- Give a brief introduction of N50K- that it is a programme focused on recruitment and retention of nurses, in an attempt to secure 50’000 more nurses in the NHS by 2024.

**Implementation**

4. What efforts and activities have been undertaken in your local Trust / geographical area since 2019 to recruit or retain nurses?  Have you seen any changes to the way recruitment and retention are usually handled since N50K was announced (2019)? Activities in relation to addressing student attrition.

Education: Consider activities and efforts to increase student applications, admissions, completion, attrition rates etc.

- Have activities focused on;  geographical area; branches/ pathways / specialties (e.g. adult, children, mental health and learning disability, community,); skills; age groups; demographics?
- What has been the main objective of the activities? (i.e. recruitment and/or retaining students)

5. How do these differ to activities and efforts that might have taken place pre-2019?

- target audience, frequency of activities, intensity of activities

6. Who has been involved in the organization or participation of these activities?

- Organising: who from national, regional, local organisations
- Participating: who are the activities / efforts targeted at (e.g., different workstreams)

7. What resources have been required to deliver these efforts and activities, and have you had the resources required?

Yes:

- What have they been? Funding, human resources (new posts), other organisationsl support?

No

- What resources would you need to delivery successful recruitment and retention activities?

8. Do you know if any efforts or activities to recruit / retain nurses were stopped / discontinued? Why was this the case?

**Impact**

9. In your Trust / local area have you seen any impact of these activities on the recruitment and retention of nurses?

- Increase in nurses (generally, of one socio-demographic group specifically?)
- Increase in staff morale / wellbeing / less staff turnover.

Education: Consider admissions / competition rates

Yes:

- What have these impacts been? How has recruitment and retention varied?

No:

- Why do you think there has been no impact?

All:

- What other factors have affected the recruitment and retention of nurses?

10. How are the efforts around recruitment and retention of nurses monitored and evaluated?

Education: Think about if /how student admissions, completion etc. are monitored.

 Who is responsible for this?

- Do you have the data needed to effectively monitor / evaluate recruitment and retention of nursing students?
- Is there anything missing, what would help you to monitor and evaluate efforts?

**What could be improved?**

11. If you could have 30 minutes with the health secretary – what would you say is the priority for the nursing workforce.

- What should be included in the workforce strategy to help expand the nursing workforce in the NHS?

(thinking about all stages of the pipeline, and all possible sources - from nursing student recruitment through to retirement?

- Do you have anything else to add? Is there anything you want to comment on, to say that I have not asked?

**Registered Nurses**

**N50K Programme**

1. Have any of you heard of the ‘N50K programme’?

Yes:

- What is your understanding of it?
- What are the programme’s objectives?

No:

- Give a brief introduction of N50K- that it is a programme focused on recruitment and retention of nurses. Aims to secure 50,000 more nurses in the NHS by 2024.

**Implementation**

2. In the last 3 years, have you experienced any efforts or activities that have been targeted at improving your working conditions or wellbeing?

- Can you tell me more about these? What did they involve? Who did they involve?
- How did they make you feel? Do you feel more valued/ better as a result of these?

3. Has your organisation consulted with you to find out what you need or want in your workplace?

Yes:

- When was this? How was it carried out? How do you feel about the level of employer consultation?

All:

- What would you like to see happen in your organisation to improve your working conditions / wellbeing?

4. What efforts and activities have been organised by your organisation to recruit nurses?

- How did you come to work here? What was the recruitment process you experienced? How did you feel about the process?

**Impact**

5. Over the last 3 years, have you experienced any changes in the workplace, thinking in terms of how it feels to work here?

- How does it feel to be working in your department / ward / setting - has this changed over recent years? What has led to you feeling this way?

6. Thinking about the recruitment and retention of nurses over the last three years, have you noticed differences in recruitment and retention in your Trust?

- Are more people joining?
- Do you or your colleagues discuss leaving the Trust or profession, has the view on this changed at all in recent times?
- What factors might have affected the Trust’s recruitment and retention of nurses?
- What do you see as the key challenges in nurse recruitment? What have you noticed in your workplace?

**How to improve**

Imagining now:

10. If you could have 30 minutes with the new health secretary – what would you tell them the priority is should be in terms of the health workforce?

- What should be included in the workforce strategy to help expand the nursing workforce in the NHS?

(thinking about all stages of the pipeline, and all possible sources - from nursing student recruitment through to retirement?

11. Do you have anything else to add? Is there anything you want to comment on, to say that I have not asked?

**Student nurses**

**N50K Programme**

1.	Have any of you heard of the ‘N50K programme’?

Yes:

- What is your understanding of it?
- What were its objectives?
- Where did you come to learn of this? (i.e. placements/education training etc.)

No:

- Give a brief introduction of N50K- that it is a programme focused on the recruitment and retention of nurses, aims to secure 50,000 more nurses in the NHS by 2024.

**Implementation**

3. Thinking about your training:

- Tell me how you came to enrol with the current organisation you are training with.
- During your training time have you experienced any efforts or activities that have been targeted at improving your training experience?
- Can you tell me more about these? What did they involve? Who did they involve?
- How did they make you feel? Do you feel more valued/ better/ committed as a result of these?

4. Thinking about your clinical placements: have you experienced any efforts or activities that have been targeted at improving the working conditions for all staff /  improving your training experience?

- Can you tell me more about these? What did they involve? Who did they involve?
- How did they make you feel? Do you feel more valued/ better as a result of these?

5. Has your University or Trust (where you undertake clinical placement) consulted with you to find out what you need or want as you train, or what you want from a workplace?

Yes:

- When was this? How was it carried out? How do you feel about the level of consultation?

All:

- What would you like to see happen at your University / clinical placement Trust to improve your study, working conditions and wellbeing?

6. Have you experienced any recruitment fairs / any activities from Trusts to recruit you once you have qualified?

- Can you tell me about these? How were they? How did you feel?
- What are you looking for when considering future employment? Main considerations.
- What recruitment strategies would you respond best to? Have you experienced any of these?

**Impact**

7. Do you feel any of the activities you’ve spoken of have had an impact on the recruitment or retention of nurses either in your Trust or fellow student nurses in the University?

8. When on placements have you or your colleagues experienced any changes in the workplace, thinking in terms of how it feels to work in the Trust?

- How does it feel to work on the wards / in placement settings - has this changed over the time you have been on clinical placements?

9. As nurses in training have you experienced any challenges in training or clinical placements that have made your training a little more difficult?

- Has anything led you to question continuing?

10. As a nurse in training have you experienced anything that has supported your training or clinical placements?

- Tell me more about this. What was it? How did it make you feel?

Imagining now:

11. If you could have 30 minutes with the health secretary – what would you say should be included in the workforce strategy to help expand the nursing workforce in the NHS, thinking specifically about what your training has been like, what would you want them to know, what should their key priorities and strategies be?

12. Do you have anything else to add? Is there anything you want to comment on, to say that I have not asked you?
