# Supplementary material for: Has a national programme to expand the nursing workforce in England been a success? A mixed methods study
Source: Int J Nurs Stud Adv. 2026 Nov 5;11:100735. doi: 10.1016/j.ijnsa.2026.100735 (PMC13770563; doi:10.1016/j.ijnsa.2026.100735)
Supplement: Supplementary file 2 [file mmc2.docx]

Supplementary tables

Table S1. N50k programme targets and areas of key interventions / activities for domestic supply

| Area of activity | Examples of key activities |
| --- | --- |
| *Increase undergraduate numbers by additional 3,700 by March 2024 and 2,400 full-time equivalent post graduate numbers by 2025* | |
| Increase course enrolment via national campaign | Promote career prospects; promote flexible / online courses; target diverse populations and career switchers |
| Increase placement capacity | Accurately estimate and monitor; increase higher education institutions/NHS coordination |
| Increase and support higher education institution capacity | Support struggling higher education institutions; assess potential demand and capacity |
| Improve student financial support | Learning support fund (5-8k) for all nursing degree students |
| Improve course flexibility | Blended learning to develop alternative routes to nursing degree |
| *Reduce pre-registration attrition from 11% to 7.7% by 2024 (optimistic target between 1,592 - 2,955 full-time equivalent)* | |
| Improve measurement | Agree attrition definition; measurement toolkit; assess Covid impact |
| Improve financial support | Streamline payment processes; advance travel payments |
| Improve sharing best practice | Identify / address higher education institution outliers e.g., staff/student ratios. |
| Improve clinical placements | £8.2m towards an additional 7,000 placements. Regional support, supervisor toolkit; map quality against framework |
| Improve online learner support | Support remote learners e.g., enhanced pastoral support |
| Improve education / NHS transition | Career information; improve tracking of first job destination |
| *Increase apprenticeships by additional 1,400 full-time equivalent by December 2025* | |
| Improve employer readiness | Map employer start dates; toolbox - career pathways, training needs |
| Increase engagement | Senior nurses as ambassadors; ensure higher education institution and Nursing and Midwifery Council readiness |
| Improve campaigns | Harness schools and college campaigns |
| Increase support to healthcare support workers | Career support / advice; identify those with ambition to progress |
| Increase nurse associate to registered nurse conversion | Ensure similar financial offer to apprenticeship offer; promotion. |
| *Increase return to practice by 6,000 Full-time equivalent by 2025* | |
| Support temporary registrants | Information booklet about routes to return to permanent register |
| Improve nationwide campaigns | Personal outreach to potential returners re financial support |
| Support Return-to-Practice course completion | e.g., where courses suspended in Covid; online learning |

Table S2.  N50k programme targets and areas of key interventions / activities for international recruitment

| Area of activity | Examples of key activities |
| --- | --- |
| N50k target: recruit additional 18,400 internationally educated nurses by March 2024 | |
| Improve marketing | Develop NHS as ‘destination of choice’; standardise NHS offer |
| Offer financial incentives to trusts | £10 million in 2020; subsequent funding dependant on success |
| Improve / standardise experience | Pastoral support; masterclasses; cultural awareness guide |
| Improve provider collaboration | Reduce competition, share expertise, spread resource / cost burden |
| Improve regional teams support | Share resources; identify trusts needing more support; facilitate collaboration; identify / escalate issues with NMC/Home Office |
| Accelerate development support | Recognise experience e.g., band 6+ roles; leadership programme |
| Specialist role recruitment support | Additional financial support e.g., community, mental health nursing |
| Pilot refugee nurse support | Support refugees with nursing background to resume career in UK |
| Improve entry processes | Increase Objective Structured Clinical Examination (OSCE) capacity; ensure visa challenges mitigated |
| Ensure trust ethical recruitment | Adherence to UK Code of Practice |
| Support diaspora organisations | Enhance pastoral offer; expand membership; provide grants |
| Government-to-government agreements | New supply routes to facilitate recruitment from more countries |

Table S3.  N50k programme retention target and areas of key interventions / activities for nurse retention

| Area of activity | Examples of key activities |
| --- | --- |
| N50k optimistic target: reduce domestic leaver rate from 6.28% to 5.63% and international leaver rate from 9.2% to 7.2% (between 7,308-7,508 full-time equivalent nurses (4,478 domestic and 2,620 non-UK) | |
| Analysis to target interventions | Target nurses most at risk of leaving; intensive support for worst performing trusts |
| Supporting new starters | Regional preceptorship leads; legacy mentoring to new starters; |
| Support flexible working | Best rostering practice; masterclasses; flexible working discussion in annual appraisals |
| Improve health and wellbeing support | Mental health support e.g., self-help apps, expand occupational health; improve e.g. workload, unnecessary bureaucracy; support trusts with high sickness/turnover |
| Inclusive / compassionate culture | Update of national freedom to speak up and whistleblowing policies; engage trusts in ‘just and learning’ culture programme |
| Pay and reward (overall pay was not an N50k intervention) | Top of band retention payments; review retire and return pension rules; convert bank only into substantive roles; educate staff on value of NHS pay/non-pay offer; information about pensions |
| Learning and development | New, online programmes for leaders/managers; set out guidance for leaders to foster compassionate, inclusive leadership |
| Equality, diversity and inclusivity | Review Covid impact on vulnerable staff groups; develop specific support for ethnic minority colleagues; good practice workshops; monitor staff survey e.g., bullying, equal opportunities |
